# Seasonal Effects on Great Ape Health: A Case Study of Wild Chimpanzees and Western Gorillas

**DOI:** 10.1371/journal.pone.0049805

**Published:** 2012-12-05

**Authors:** Shelly Masi, Sophie Chauffour, Odile Bain, Angelique Todd, Jacques Guillot, Sabrina Krief

**Affiliations:** 1 Muséum national d'histoire naturelle, Département Hommes, Natures, Sociétés UMR 7206 Éco-anthropologie et Ethnobiologie, Paris, France; 2 Ecole Nationale Vétérinaire d'Alfort, Parasitologie, UMR Anses, Enva, Upec BIPAR, Maisons-Alfort, France; 3 Muséum national d'histoire naturelle, et CNRS, Parasitologie comparée, UMR 7205 OSEB, Paris, France; 4 Dzanga-Sangha Protected Areas, WWF, Bangui, Central African Republic; 5 Projet pour la Conservation des Grands Singes, Paris, France; Université Pierre et Marie Curie, France

## Abstract

Among factors affecting animal health, environmental influences may directly or indirectly impact host nutritional condition, fecundity, and their degree of parasitism. Our closest relatives, the great apes, are all endangered and particularly sensitive to infectious diseases. Both chimpanzees and western gorillas experience large seasonal variations in fruit availability but only western gorillas accordingly show large changes in their degree of frugivory. The aim of this study is to investigate and compare factors affecting health (through records of clinical signs, urine, and faecal samples) of habituated wild ape populations: a community (N = 46 individuals) of chimpanzees (*Pan troglodytes*) in Kanyawara, Kibale National Park (Uganda), and a western gorilla (*G. gorilla*) group (N = 13) in Bai Hokou in the Dzanga-Ndoki National Park (Central African Republic). Ape health monitoring was carried out in the wet and dry seasons (chimpanzees: July–December 2006; gorillas: April–July 2008 and December 2008–February 2009). Compared to chimpanzees, western gorillas were shown to have marginally greater parasite diversity, higher prevalence and intensity of both parasite and urine infections, and lower occurrence of diarrhea and wounds. Parasite infections (prevalence and load), but not abnormal urine parameters, were significantly higher during the dry season of the study period for western gorillas, who thus appeared more affected by the large temporal changes in the environment in comparison to chimpanzees. Infant gorillas were the most susceptible among all the age/sex classes (of both apes) having much more intense infections and urine blood concentrations, again during the dry season. Long term studies are needed to confirm the influence of seasonal factors on health and parasitism of these great apes. However, this study suggest climate change and forest fragmentation leading to potentially larger seasonal fluctuations of the environment may affect patterns of ape parasitism and further exacerbate health impacts on great ape populations that live in highly seasonal habitats.

## Introduction

Understanding the interplay between ecosystem modification and the probability of disease transmission is crucial in predicting changes in infectious disease patterns, reducing health risks for fauna and local people, and assisting wildlife conservation management [1], [2]. Given their phylogenetic relatedness, wild populations of non-human primates are an indicator species for monitoring emerging pathogens, providing crucial information on infectious diseases that may also affect humans (e.g. [1], [3]–[11]).

Usually, assessing intestinal parasite load of wild primates is considered a fairly acceptable proxy for evaluating health status since intestinal parasites may be detected in all species and severe pathogenic infections may reduce host fitness. However, some intestinal parasites can either be symptomatic (e.g. mucosal inflammation, diarrhoea, ulceration, and weight loss) or asymptomatic depending on host general health status, and the level of infection and parasite species [12]. Therefore, a combination of different non-invasive techniques (coproscopy, urine analysis and monitoring of clinical signs) is a more powerful diagnostic tool for assessing health in wild primate populations [3], [6], [9] than one technique alone.

Among the potential interacting factors, environmental variables (e.g. habitat characteristics, climate) can be important influences on parasite community composition and the level of parasitic infection in wild primate populations (e.g. [13], [14]). Furthermore, habitat degradation and disturbance, increased local human density and spatial proximity (and consequently that of domestic animals) may result in drastic microhabitat and microclimate changes leading to higher prevalence and diversities of intestinal parasitic infections [14]. Additionally, seasonal patterns of parasite infections have also been detected in some primate species (e.g. [15], [16] but see also [17] and are likely caused either by changes in parasite life history - e.g. fecundity and development - as shown in domestic animals (sheep: [18], pig: [19]), or by host seasonal diet changes, typical for some primates facing periods of fruit scarcity [20]–[22]. In fact, and particularly during such periods, intestinal parasites may intensify malnutrition reducing host ability to absorb nutrients by altering host digestive efficiency or by competing for food supply [23]. Moreover, dietary stress (e.g. low protein to fibre ratio in the diet occurring during period of fruit scarcity) may directly contribute to increased intestinal parasite infections and lead to the development of multiple infections (e.g. sheep: [24], ruminants: [25], colobus monkeys: [26]). The confounding interaction of such factors is difficult to assess even though the synergetic effect may impact host survival and reproduction leading to population declines [26]–[28].

Besides environmental and nutritional factors, social and species-specific behaviors (e.g. grooming, inter-individual proximity, co-foraging and ranging) likely also affect patterns of disease transmission. In social living primates, intestinal parasite levels may be density-dependent and thus positively associated with group size [29], [30], although this pattern remains controversial [26]. Specific primate behaviours such as changing sleeping sites daily and social grooming may play both positive (by reducing contact with old faeces, removing ectoparasites, limiting insect biting vectors of parasites) and negative roles (by increasing individual proximity) in parasite transmission [31]–[33]. Additionally, parasite infections may be buffered by the ingestion of secondary metabolites alongside daily diet or by specific self-medication behaviours such as (rough) leaf swallowing (e.g. [6], [7], [15], [34]–[40]). Health status may thus also be affected by sex/age class, rank and social stress [41], [42]. Primate infants (and pregnant females) are typically more susceptible risk to disease, with higher prevalence of homoxenous parasites (e.g. strongylid, threadworms) than adults who have developed immunity after repeated exposures ([43]–[47]; but see [48]). With regards to social stress, in high ranking chimpanzees, increased intestinal parasite load is associated with higher testosterone levels [49]. Our closest relatives, the great apes, are particularly sensitive to infectious diseases due to a slow reproductive rate and prominent infant mortality (e.g. [3], [10], [50]–[55]).

No baseline data exist on urinary parameters of wild western gorillas (*Gorilla gorilla*; for mountain gorillas – *Gorilla b. beringei* - see [56]) but for wild chimpanzees (*Pan troglodytes*) systematic long-term data collection of urinary parameters are already available [6], [57]. Although characterized by a less cohesive social system (fission-fusion societies in chimpanzees vs. one-male harems in western gorillas), chimpanzees show higher social tolerance and interactions, including social grooming which is almost absent in western gorillas [58]–[61]. Moreover, chimpanzees and western gorillas respond differently to seasonal changes in fruit availability even if they share extensive flexibility and a high degree of frugivory [21], [22], [60], [62]. While chimpanzees remain mainly frugivorous even when fruit becomes scarce, western gorillas can switch to a more herbivorous diet as they are physiologically better adapted to digest fiber [60], [62]–[66]. During periods of fruit abundance both species improve dietary quality (in terms of food energy content for western gorillas and macronutrients for chimpanzees) but their overall energy intake is not affected by changes in fruit availability [22], [67]–[69]. Previous studies have shown that the absence of seasonal nutritional stress in mountain gorillas makes the effect of intestinal parasite infections negligible [70]. In chimpanzees higher infection of some intestinal parasites has been found to occur during wet seasons ([6], [16], [71] but see [72]). When fruit is scarce in drier seasons, the higher consumption of vegetative plant parts, particularly by western gorillas, provides a higher concentration of potentially anti-parasitic secondary compounds which may help limit parasite infections and reduce clinical signs [7], [15], [17], [34]–[37], [40], [73], [74]. Whilst ingestion of such therapeutic compounds may seem to be more advantageous for western gorilla health, chimpanzees may instead be more reliant on specific ingestion of certain bioactive plants which they appear to consume more frequently than western gorillas [61]. The impact of these social and ecological traits on great ape health remains unclear.

This study aims to compare and investigate changes in the health of wild chimpanzees and western gorillas across time periods and age/sex classes. Even though the results of this study need to be interpreted with caution, being based on a single wet and dry season from one group of each species living in non-sympatric populations, some tenable predictions can be made. If in chimpanzees, compared to gorillas, more frequent social contact plays an important role in parasite transmission we predict 1) higher infection rate (prevalence and intensity) for chimpanzees compared to western gorillas. Alternatively, if in gorillas, compared to chimpanzees, greater seasonal changes in dietary composition has a strong influence on health, the opposite pattern can be predicted, i.e. higher infection rate in gorillas. We also predict 2) temporal variation in infection rates (as detected by intestinal parasite prevalence and load and abnormal urine parameters) especially for western gorillas since they are more affected by seasonal habitat changes. Based on previous ape parasitological studies on seasonality (available for chimpanzees only [6], [16], [71]), we may expect 3) higher infection rates during the wet season of the study periods in particular, and 4) as for other primates [43]–[47], in infants of both apes with respect to the other age/sex classes.

## Methods

Permits and approvals for field work and biological sampling were delivered by the Uganda Wildlife Authority, the Uganda National Council for Science and Technology, and the Ministries of Education and Water and Forests, Fishing and Hunting of the Central African Republic. This research adhered to ethics, protocols and legal requirements of both African countries.

### Study site and groups

Data were collected on the Kanyawara community of eastern chimpanzees (*Pan troglodytes schweinfurthii*) in Kibale National Park, Uganda, by SK and her field assistants from July to December 2006. The Park, covering 795 km^2^, is home to approximately 1000 chimpanzees and includes elements of lowland tropical rain forest, mountain rain forest and mixed deciduous rain forest (altitude: 1500 m; mean annual rainfall: 1719 mm between 1990 and 2005; human population density around the park up to 335 hab./km^2^ [75], [76] Chapman unpublished data in [77]). Kibale has two rainy seasons generally from March to May and from September to November with a drier season in between, but this pattern is unpredictable with large inter-annual variability and local rainfall variability [78]. Kanyawara's community of chimpanzees was stable during the study period and included 46 individuals: 9 adult males, 11 adult females, 9 subadults (3 males, 6 females), 10 juveniles (7 males, 3 females) and 7 infants (3 males, 4 females); age-classes following [79]).

Data on western lowland gorillas (*G. g. gorilla*) were collected by S.M. at Bai Hokou from May to July 2008 and from December 2008 to February 2009 in Dzanga-Ndoki National Park, Central African Republic (western gorilla density of Dzanga sector, 495 km^2^: 1.6 indiv./km^2^ – [80] and Todd, unpublished data). The largest human settlement, Bayanga is approximately 30 km away from Bai Hokou (approx. 3,925 habitants of the 6,188 in the Reserve buffering the National Park [81]). Bai Hokou includes both primary and secondary lowland tropical rain forest (altitude: 340–615 m) and has one rainy season from April to November with a drier season from December to February/March (rainfall<80 mm per month; mean annual rainfall: 1700 mm). During the study period the study group of gorillas, the Makumba group, was composed of 11–13 individuals: one silverback, three adult females, one blackback, zero to two subadult females (who emigrated from their natal group), three juveniles and three infants; age/sex classes following [82]. Apes were observed daily by research teams at a distance of at least 5–7 m.

To monitor seasonal changes in meteorological data, temperature and percentage humidity were recorded in the forest twice per day with a portable digital thermo-hygrometer (7:30–8:30 a.m. and 4:30–6 p.m.). Rain data were obtained from another Kanyawara study [77] and recorded daily at Bai-Hokou.

### Clinical signs

Clincal signs were recorded daily during 15 min individual focal follows for the maximum possible number of chimpanzees present in the party during the study period (on average 13 individuals monitored per day, 1725 15 min sessions, N_days_ = 132 days) and for gorillas during focal follows of all group members ([83]; 188 days, 714 hours of focal follows). We monitored respiratory, reproductive, locomotion, urinary and digestive functions and recorded all cases of any clinical signs including sneezing, nose discharge, coughing, breathing problems (dyspnea), increased respiratory rhythm, swelling of body parts, wounds, abscesses, ophthalmic lesions, limping, vomiting, abnormal fecal consistency, increased or decreased defecation/urination frequency, lethargy, increased water intake (polydipsia), anorexia.

### Non-invasive biological sampling

We endeavored to collect biological samples daily from different identified individuals to balance sample collections across group members and across the months of the study period (Figure S1). Coproscopic examination was carried out on 203 stool samples from 31 chimpanzees and 182 samples from 16 gorillas (per season: Table 1). Faecal samples (N = 3) from three lone silverbacks ranging around the Makumba group were collected opportunistically and added to the analysis to increase male sample size in gorillas. Immediately following defecation, individual fecal samples were inspected for consistency (liquid, soft/pasty, solid-normal, dry/hard following Krief et al. [6]) and presence of whole leaves (as evidence of leaf swallowing) and/or macroscopic parasites that were stored in ethanol and identified based on morphological description at species level by O.B. [9]. Two grams of fresh stools were preserved for coproscopy analysis in 18 ml of 10% formalin saline solution [6]. Smears of all samples were made with 50 ml of the suspension and were microscopically examined by S.C. at the department of Parasitology, Alfort Veterinary College (France). According to size, colour and shape, parasite eggs, larvae and adults as well as ciliates were counted and identified at the genus or species level. Eggs per gram (EPG) or adult counts were corrected according to faecal consistency (dry faeces: corrected parasite load CPL = EPG count*0.5, soft: CPL = EPG count*2, liquid stools: CPL = EPG*3; [7], [84]).

**Table 1:** Seasonal subdivision of the study period and biological sample sizes.

	SPECIES	SEASON	
		Wet	Dry
**Study Period**	*Gorilla gorilla*	May–Jul 08	Dec 08–Feb 09
	*Pan troglodytes*	Oct–Dec 06	Jul–Sep 06
**Temperature (Celsius)**	*Gorilla gorilla*	24±1 (17–33)	26±2 (16–34)
	*Pan troglodytes*	18±2 (13–24)	18±2 (13–24)
**Humidity (%)**	*Gorilla gorilla*	82±8 (62–96)	60±10 (39–82)
	*Pan troglodytes*	88±8 (69–98)	85±11 (57–98)
**Rain (mm/month)**	*Gorilla gorilla*	>80	<80
	*Pan troglodytes*	>80	<80
**# Fecal samples**	*Gorilla gorilla*	70	105
	*Pan troglodytes*	90	113
**# Urine samples**	*Gorilla gorilla*	51	126
	*Pan troglodytes*	82	78

In parenthesis: minimum and maximum values.

Individual urine samples were collected and analyzed within 30 min as previously described by Krief et al. [6]. Only urine uncontaminated by feces and soil were collected. A total of 337 samples were collected and tested including 160 urine samples from chimpanzees (61 samples of adult males, 53 of adult females, 18 of subadult males, 11 of subadult females, 15 of juveniles and 2 of infants) and 177 samples from gorillas (29 of adult males, 53 of adult females, 23 of subadult males, 13 of subadult females, 42 of juveniles and 19 of infants; samples per seasonal period of the study: Table 1). Abnormal levels of a number of urine parameters were evaluated via commercial dry reagent dipsticks (Multistix 10 SG Bayer): pH, specific gravity, glucose, proteins, nitrites, ketones (by-product of fat metabolism), hemoglobin (hemolysis indication), bilirubin (hemoglobin degradation product), urobilinogen (bilirubin degradation product) and quantity of leukocytes.

### Data analysis and statistics

We considered 1) the percentage of faecal samples positive for each parasite species or genus – as a proxy for parasite infection prevalence, and 2) the mean corrected parasite load (mean CPL): the arithmetic corrected mean (mean abundance) including infected and non-infected samples – as a proxy for parasite infection intensity. The study periods were subdivided into dry and wet seasons on the base of meteorological data, collected in the field for Bai Hokou and from the literature for Kanyawara, and consequent changes in ape diet (Table 1). To investigate the effect of host populations (chimpanzees and gorillas), time periods (dry and wet season of the study periods) and age/sex classes on intestinal parasite load, a three-way ANOVA with replicates (individual measures) was performed by the statistical package STATISTICA 6. All factors were treated as fixed factors. To investigate the effect of the same three factors on the percentage of positive samples for each parasite species (prevalence) we first obtained the mean percentage of positive samples per individual per time period, and then we performed a repeated measure ANOVA including as “between subject factors” population and age/sex classes and as “within subject factor” the time periods. To avoid missing cells, individuals with no faecal samples collected in one time period were excluded from the analysis. Both ANOVAs were fully crossed, and data were marginally unbalanced, thus to test independence of sample size we used Type III sums of squares. Data for three-way ANOVAs were squared root transformed to fulfil the assumptions of homoscedasticity and their normality inspected by Levene's test and visually by p-p and q-q plots as well as residuals plotted against fitted values. In the text, only significant interactions among the three tested factors are displayed. Within host population comparisons (e.g. for parasites typical of one ape population only) were carried out using Wilcoxon Test for comparing time periods, and Kruskal-Wallis H-test for comparisons among the age/sex classes.

## Results

### General body condition and clinical signs

**Figure 1:**
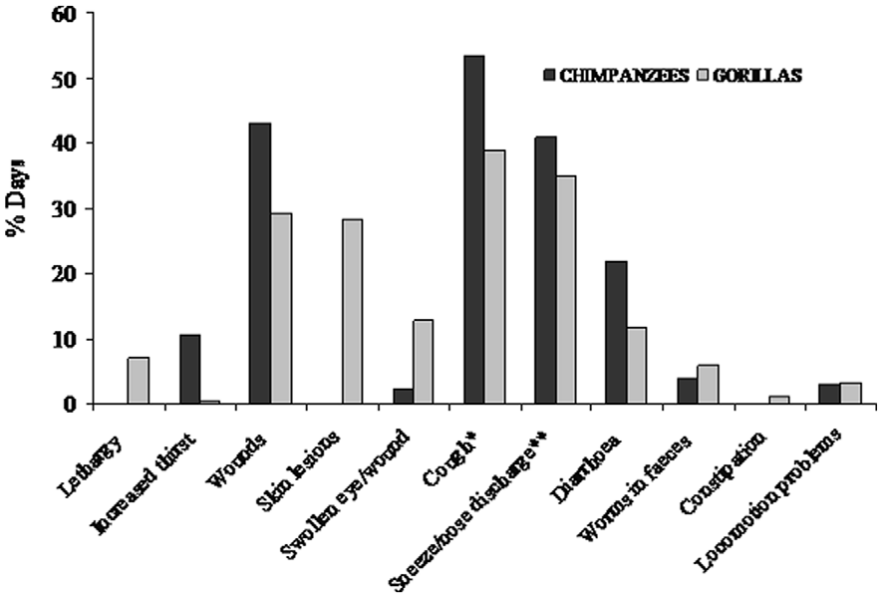
Summary of general body conditions and clinical signs. Percentage of days with at least one individual with each symptom for chimpanzees (N = 132 days) and gorillas (N = 188 days). *Wounds* consisted of bites or cuts caused by falls from trees, intra and inter (only for gorillas, but caused by vegetation) group aggressive interactions. *Skin lesions* consisted in itchy white spots (sometimes with little crusts) on body and face of all group members, possibly produced by either a fungus or ringworms. *Worms in faeces*, in chimpanzees two *Bertiella* sp segments associated with whole leaves of *Aneilema aecquinoctiale*. *Diarrhoea*, in gorillas 2 cases caused by stress related during inter-group interactions, one severe case from a 2 year-old infant lasting two days, mild diarrhoea during dry season (N = 9). Asterisks indicate a different sample size for chimpanzees: *N = 127, ** N = 125.

The most common clinical signs observed for the two species were coughs and sneezes as shown in the comparative summary for each study group in Figure 1. No differences were found between the two ape populations in the proportion of days with respiratory symptoms (data controlled for number of individuals observed per day; Mann-Whitney U Test, N_Chimpanzees_ = 131, N_Gorillas_ = 188, cough: z = 1.08, P = 0.277; sneeze/nose discharge: z = −0.42, P = 0.668), but chimpanzees had higher occurrence of days with diarrhoea and wounds with respect to gorillas (N_Chimpanzees_ = 131, N_Gorillas_ = 188, diarrhoea: z = 2.64, P = 0.008; wounds: z = 2.16, P = 0.030). Eye lesions were rare in chimpanzees but quite frequent in gorillas and skin lesions were observed only for gorillas (see legend of Figure 1 for description). Whole leaves of *Aneilema aecquinoctiale* and *Rubia cordifolia* were recovered in eight chimpanzee stools with (N = 2) and without macroparasites (N = 6); for gorillas no intact hairy or rough leaves were ever observed in faeces nor any leaf swallowing behaviour (Bai Hokou long term data).

### Coproscopic examination

A number of parasite species and non-parasitic symbiotic ciliates were found in both host study populations (Table 2): nematodes such as the large eggs and adults of strongyles (family Strongylidae, 50×90 µm), probably all *Oesophagostomum sp.* [9], adults and larvae of *Probstmayria gombensis* (Atractidae), the cestode *Bertiella studeri* (Anoplocephalidae), and the ciliates of the genus *Troglodytella*. Other species were found only in a single ape population. Additionally, chimpanzee samples included eggs of one other parasite, *Strongyloides fullebornii* (Strongylidae) and a small entodiniomorph ciliate (22×20 µm), while gorillas samples included a number of small unidentified heterogeneous strongylid eggs (40×80 µm), and two more nematodes, specifically, eggs of *Mammomonogamus sp.* (Syngamidae) and one adult (female) of *Protospirura muricola* (Spiruridae). This last nematode was identified by morphological description together with two females of *Oesophagostomum stephanostomum* from gorilla feces.

**Table 2:** Summary of coproscopy (a–b) and macroscopic results (c) for both chimpanzees and western gorillas.

		AGE/SEX	ALL	ADULT	ADULT	SUB-ADULT	SUB-ADULT	JUVENILES	INFANTS	UNKNOWN
INFECTION INDEX		CLASSES	INDIVIDUALS	MALES	FEMALES	MALES	FEMALES			INDENTITY
**A)**		**SPECIES**								
**# OF SAMPLES (# INDIVIDUALS)**		***Gorilla gorilla***	178 (16)	36 (4)	51 (3)	16 (1)	14 (2)	39 (3)	22(3)	2 (2)
		***Pan troglodytes***	205 (38) ♀: 116 (22) ♂: 89 (16)	59 (9)	68 (11)	19 (3)	31 (5)	19(6) ♀: 15 (4) ♂: 4 (2)	9 (40) ♀: 2 (2) ♂: (2)	-
**% POSITIVE SAMPLES**	**All parasites** (ciliates excluded)	***Gorilla gorilla***	91 (100)	67 (100)	82 (100)	63 (100)	79 (100)	82 (100)	96 (100)	100 (100)
**(% POSITIVE INDIVIDUALS)**		***Pan troglodytes***	55 (32)	57 (89)	55 (73)	50 (100)	55 (100)	38 (71)	33 (31)	-
	**Nematodes**									
	Strongyl large eggs *Oesophagostomum sp.* (50×90 µm)	***Gorilla gorilla***	73 (100)	61 (50)	78 (100)	65 (100)	57 (100)	77 (100)	91 (100)	100 (100)
		***Pan troglodytes***	46 (77)	44 (89)	52 (64)	37 (100)	55 (100)	♀: 27 (75) ♂: 75 (100)	♀: 0 ♂: 29 (50)	-
	Strongyl small eggs (40×80 µm)	***Gorilla gorilla***	49 (94)	42 (50)	47 (100)	18 (100)	50 (100)	56 (100)	76 (100)	50 (50)
	*Strongyloides fulleborni*	***Pan troglodytes***	8 (42)	6.8 (22)	7 (36)	11 (67)	10 (60)	♀: 0 ♂: 0	♀: 50 (50) ♂: 29 (50)	
	*Probstmayria gombensis* (adult+young)	***Gorilla gorilla***	10 (64)	8 (25)	12 (100)	18 (100)	21 (100)	3 (33)	10 (33)	0
		***Pan troglodytes***	16 (58)	20 (67)	13 (55)	11 (67)	10 (40)	♀: 7 (25) ♂: 0	♀: 0 ♂: 43 (50)	-
	*Mammomonogamus sp* eggs	***Gorilla gorilla***	22 (79)	31 (50)	37 (100)	6 (100)	14 (100)	10 (67)	19 (33)	0
		***Pan troglodytes***	0	0	0	0	0	0	0	-
	**Cestodes**									
	*Bertiella studeri* eggs	***Gorilla gorilla***	0	0	0	0	0	0	0	0
		***Pan troglodytes***	4 (18)	2 (11)	7 (36)	0	10 (40)	♀: 0 ♂: 0	♀: 0 ♂: 0	-
	**Protozoa**									
	*Troglodytella spp.*	***Gorilla gorilla***	31 (27)	33 (69)	28 (67)	35 (100)	36 (100)	44 (100)	24(67)	0
		***Pan troglodytes***	80 (100)	73 (100)	77 (100)	63 (100)	65 (100)	♀: 93 (100) ♂: 100 (100)	♀: 100 (100) ♂: 100 (100)	-
	Small Entodiniomorphs	***Gorilla gorilla***	0	0	0	0	0	0	0	0
		***Pan troglodytes***	7 (42)	6 (44)	9 (46)	5 (33)	6 (20)	♀: 0 ♂: 25 (50)	♀: 0 ♂: 14 (50)	-
**B)**										
**MEAN CORRECTED PARASITE LOAD**	**Nematods**									
**(RANGE)**	Strongyle large eggs (eggs/g) (50×90 µm)	***Gorilla gorilla***	368.5 (0–3100)	280.6 (0–1200)	290.2 (0–1100)	237.5 (0–700)	114.3 (0–300)	505.1 (0–3100)	709.1 (0–3100)	4.5 (3–6)
		***Pan troglodytes***	195.3 (0–3800)	128.1 (0–1200)	292.7 (0–3100)	57.9 (0–200)	293.5 (0–3800)	♂: 150 (0–300) ♀: 36.7 (0–200)	♂: 157.1 (0–600) ♀: 0	-
	Strongyle small eggs (eggs/g) (40×80 µm)	***Gorilla gorilla***	184.2 (0–2400)	91.7 (0–600)	117.6 (0–600)	43.8 (0–300)	164.3 (0–700)	233.3 (0–1400)	518.2 (0–2400)	3.0 (0–6)
	*Strongyloides fulleborni*	***Pan troglodytes***	15.0 (0–400)	19.7 (0–400)	9.6 (0–200)	26.3 (0–300)	19.4 (0–400)	♂: 10.0 (0–100) ♀: 0.0	♂: 14.3 (0–100) ♀: 50.0 (0–100)	-
	*Probstmayria gombensis* (adult+larvae/g)	***Gorilla gorilla***	20 (0–900)	8.3	13.7 (0–2)	18.8 (0–2)	28.6 (0–2)	2.6 (0–1)	81.8 (0–4)	0.0
		***Pan troglodytes***	23 (0–400)	31.6 (0–400)	20.6 (0–300)	26.3 (0–400)	16.1 (0–300)	♂: 50.0 (0–200) ♀: 0.0	♂: 42.9 (0–100) ♀: 0.0	-
	*Mammomonogamus spp* (eggs/g)	***Gorilla gorilla***	28.1 (0–300)	30.6 (0–200)	47.1 (0–300)	6.3 (0–100)	14.3 (0–100)	15.4 (0–300)	27.3 (0–200)	0.0
		***Pan troglodytes***	0.0	0.0	0.0	0.0	0.0	0.0	0.0	-
	**Cestodes**									
	*Bertiella studeri*	***Gorilla gorilla***	0.0	0.0	0.0	0.0	0.0	0.0	0.0	0.0
		***Pan troglodytes***	15.1 (0–1200)	6.7 (0–400)	19.8 (0–1200)	0.0	9.4 (0–200)	0.0	♂: 0.0 ♀: 0.0	-
	**Protozoa**									
	*Troglodytella spp* (ptz/g)	***Gorilla gorilla***	217 (0–9300)	127.7 (0–1400)	264.7 (0–9000)	68.8 (0–300)	378.6 (0–4500)	87.2 (0–400)	490.9 (0–9300)	0.0
		***Pan troglodytes***	2507 (0–31600)	3094.7 (0–25600)	1438.2 (0–24200)	3710.5 (0–30400)	3125.8 (0–31500)	♂: 8500.0 (100–31600) ♀: 1173.3 (0–10100)	♂:1871.4 (100–5600) ♀: 1350.0 (600–2100)	-
	Small Entodiniomorphs	***Gorilla gorilla***	0.0	0.0	0.0	0.0	0.0	0.0	0.0	0.0
		***Pan troglodytes***	85.7 (0–8800)	36.0 (0–900)	143.4 (0–8800)	10.5 (0–200)	97.0 (0–2600)	♂: 240.0 (0–2000) ♀: 0.0	♂: 285.7 (0–2000) ♀: 0.0	-
**C)**										
**MACROPARASITES**										
**# PARASITES**	**Nematodes**									
**(# INDIVIDUAL APE INFECTED)**	*Oesophagostomum stephanostomum*	***Gorilla gorilla***	0	0	0	0	0	0	0	2 (1)* associated with diarrhea for fear
		***Pan troglodytes***								
	*Protospirura muricola*	***Gorilla gorilla***	1	0	0	0	0	0	0	0
		***Pan troglodytes***								
	**Cestodes**									
	*Bertiella studeri*	***Gorilla gorilla***	8 (5)	0	5 (2)	1	0	1	0	1 (1)*
		***Pan troglodytes***	7	1	3	1	2	0	0	

* Feces either from a adult female or male, or a subadult of the Makumba group

Given the uncertainty of juvenile and infant sex in gorillas, only the average value for all individuals per each of these two classes is provided for western gorillas. Strongyl large eggs: (50×90 µ) on average, more round and fringes than the small strongyl eggs (40×80 µ) and *Strongyloides fullebornii*.

Prevalence and mean parasite load per age/sex classes per host population are summarized in Table 2. Gorillas had a significantly higher percentage of parasite-positive faecal samples (mean across age/sex classes: Chimpanzee = 50%, Gorilla = 89%, F_1,362_ = 12.52, P = 0.001), but there was no influence of either time period (dry season: Chimpanzee = 58%, Gorilla = 93%; wet season: Chimpanzee = 38%, Gorilla = 83%; F_1,362_ = 1.27, P = 0.269) or age/sex classes within and between the populations (Table 2; F_5,362_ = 0.39, P = 853).

With regard to specific parasite species, the percentage of positive samples with (large and small) strongyle eggs was twice as high in gorillas than in chimpanzees (Chimpanzee = 46%, Gorilla = 84%, F_1,29_ = 7.84, P = 0.009), and higher for both ape populations during the dry season (Chimpanzee = 52%, Gorilla = 93%) compared to the wet season (Chimpanzee = 36%, Gorilla = 73%; F_1,29_ = 4.43, P = 0.044), but did not differ among age/sex classes (F_5,29_ = 0.54, P = 0.747). However for gorillas only, the percentage of positive samples for large strongyle eggs (probably *Oesophagostomum* spp.) was higher during the dry season (84%) compared to the wet season (59%, Wilcoxon Matched Pairs Test: T^+^ = 5, N = 12, P = 0.022) and the same tendency was found for small strongyle eggs (dry season: 58%, wet season: 40%, T^+^ = 10, N = 12, P = 0.074). In chimpanzees, while *Strongyloides fulleborni* eggs (strongylid species) were also higher during the dry season (15%) compared to the wet season (2%, T^+^ = 12.5, N = 31, P = 0.038), no seasonal difference was found in the percentage of positive samples for *Oesophagostomum* spp. (dry: 45%, wet: 50%; T^+^ = 71.50, N = 27, P = 0.542).

The percentage of positive samples for *Probstmayria gombensis* did not differ between host populations (F_1,29_ = 0.05, P = 0.833), time periods (dry: Chimpanzee = 18%, Gorilla = 9%; wet: Chimpanzee = 15%, Gorilla = 12%; F_1,29_ = 0.00, P = 0.987) or age/sex classes (F_5,29_ = 0.22, P = 0.950). In gorillas only, the percentage of positive samples for *Mammomonogamus sp.* tended to be higher during the wet season (36%) compared to the dry season (14%, T^+^ = 5, N = 12, P = 0.067). The percentage of positive samples with *Troglodytella sp.* was much higher in chimpanzees than in gorillas (F_1,29_ = 36.70, P<<0.001) but no influence of time period (dry: Chimpanzee = 78%, Gorilla = 23%; wet: Chimpanzee = 82%, Gorilla = 39%; F_1,29_ = 2.06, P = 0.162) and age/sex class was found (F_5,29_ = 1.16, P = 0.355). In chimpanzees only, the percentage of positive samples for other small ciliates was higher during the dry season (11%) compared to the wet season (1%, T^+^ = 5.5, N = 31, P = 0.005).

**Figure 2:**
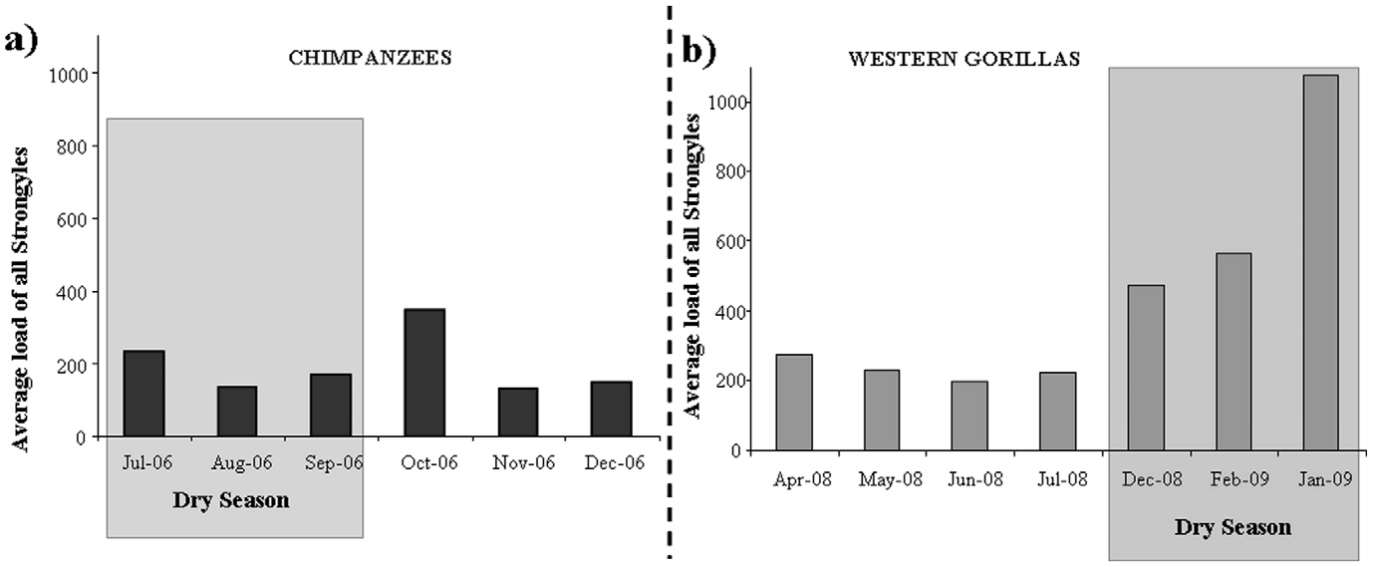
Averages per time period of strongyle load (a) in chimpanzees and (b) in western gorillas. The grey area distinguishes the months of dry season in comparison to months of wet season.

**Figure 3:**
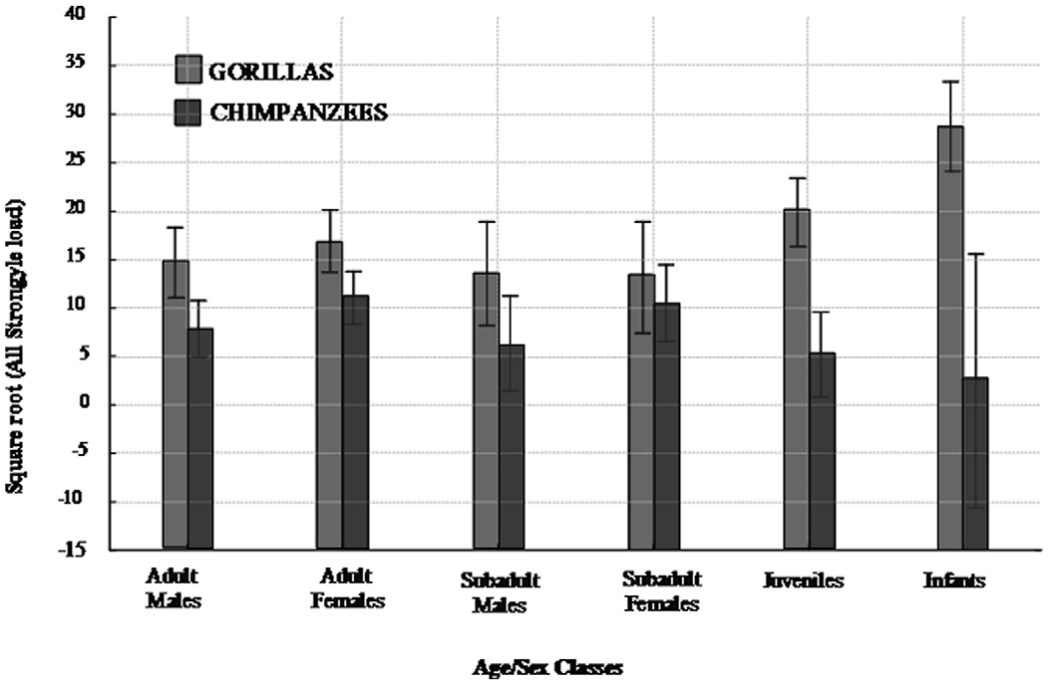
Average strongyle load across populations and age/sex classes. Vertical bars denote 0.95 confidence intervals.

The mean strongyle (all eggs) count was significantly higher in gorillas (518 Eggs per gram (EPG) [min-max: 0–4500]) in comparison to chimpanzees (210 EPG [0–3800], F_1,362_ = 45.00, P<<0.001), differed between time periods (dry: Chimpanzee = 190 EPG [0–3100], Gorilla = 726 EPG [0–4500]; wet: Chimpanzee = 236 EPG [0–3800], Gorilla = 219 EPG [0–1400]; F_1,362_ = 23.38, P<<0.001) but not between age/sex classes (F_5,362_ = 1.16, P = 0.327; Fig. 2a–b). The significant interaction between ape populations and time periods (F_1,362_ = 21.15, P<<0.001) showed that the differences were driven by gorillas “all strongyle” EPGs which were higher during the dry season with respect to the wet season and with respect to chimpanzees, whose strongyle EPGs conversely tended only to be higher during wet season (Fig. 2b, excepting *Strongyloides fulleborni* eggs which did not differ between time periods; dry: 18 [0–400], wet: 11 [0–400], T^+^ = 17.5, N = 27, P = 0.092). Ape populations also interacted with age/sex class (F_5,362_ = 3.65, P = 0.003; Fig. 3) and post-hoc analysis revealed that infant gorilla “all strongyle” egg counts were higher than all other sex/age classes (between MS = 118.28, df = 357, all P<0.001) and juvenile eggs counts were higher than those of adult males (P = 0.031) and subadults (P_Males_ = 0.038, P_Females_ = 0.027). For strongyle adults such as *Probstmayria gombensis* counts did not differ between populations (F_1,362_ = 0.18, P = 0.673), time periods (dry: Chimpanzee = 29 [0–400], Gorilla = 23 [0–900]; wet: Chimpanzee = 16 [0–200], Gorilla = 15 [0–200]; F_1,362_ = 2.02, P = 0.156) or age/sex classes (F_1,362_ = 0.27, P = 0.927). In gorillas, the number of *Mammomonogamus* eggs in gorillas likewise differed among age-sex classes (Kruskal-Wallis H-test: χ^2^ = 13.19, d.f. = 5, P = 0.022) but not between time periods (dry = 17 [0–300], wet = 44 [0–300], T^+^ = 9.00, N = 12, P = 0.110).

*Troglodytella sp.* counts were approximately ten times higher in chimpanzee faeces than in gorillas (F_1,362_ = 42.64, P<<0.001) though no effect of time periods (dry: Chimpanzee = 2519 [0–31600], Gorilla = 148 [0–9300]; wet: Chimpanzee = 2492 [0–31500], Gorilla = 317 [0–9000]; F_1,362_ = 0.26, P = 0.607), or age/sex class was found (F_5,362_ = 0.80, P = 0.553). In chimpanzee small ciliates were more abundant during the dry season (150 [0–8800]) compared to the wet season (6 [0–400], T^+^ = 2.00, N = 28, P = 0.002).

### Urine analysis

An overview of the urine analysis results for both chimpanzees and western gorillas is given in Table 3 and in Table S1. To investigate differences between ape populations, time periods and age/sex classes only the most informative urine variables were analysed: erythrocyte, leukocyte, glucose and protein concentration; the average values alongside statistics for these parameters are summarized in Table 3 and in the text only significant results are reported. **Erythrocyte** concentration (Ery/microL) differed among age/sex classes (F_5,270_ = 8.31, P<<0.001) which interacted with ape population (F_5,270_ = 5.34, P<<0.001). Post-hoc comparisons revealed subadult female chimpanzees to have higher urine erythrocyte concentrations than adult males chimpanzees (Fisher LSD: between MS = 135.17, df  = 270, P = 0.044) and similarly subadult female gorillas with respect to all other classes of gorillas (all P<<0.001 except for P_Subadult Male_ = 0.022). In addition, gorillas were found to have higher erythrocyte concentrations than chimpanzees (all P<<0.001) with the exception of subadult females. For this parameter, age-sex class interacted with time period (F_5,270_ = 3.65, P = 0.003) and during the dry season subadult females of both populations had higher urinary blood concentrations (11.82 [0.00–80.00]) compared to all other age-sex classes in any time period (all between 0.28 and 6.92 [0.00–80.00] including subadult females in the wet season) with the exception of infants during the wet season (10.00 [0.00–50.00] between MS = 135.17, df  = 270, all P<0.01). In both ape populations, infant erythrocyte concentration was higher during both time periods in comparison to the other age-sex classes except for subadult females (both time periods) and subadult males (wet season; between MS = 135.17, df  = 270, all P<0.01). **Leukocyte** concentration (Leuc/microL) was higher in gorillas than in chimpanzees (F_1,362_ = 25.16, P<<0.001) even though chimpanzees had the highest recorded concentration (500 Leuc/microL: N_Chimpanzees_ = 2, N_Gorillas_ = 0; Table S1). **Protein** concentration (g/L) was higher in chimpanzees than in gorillas (F_1,268_ = 6.65, P = 0.010).

**Table 3:** Comparative summary of average concentration and statistics for selected urine variables for chimpanzees and western gorillas.

	Species	Total Average	*ANOVA Species*	Dry Season	Wet Season	*ANOVA Season*	Adult Males	Adult Females	Subadult Males	Subadult Females	Juveniles	Infants	*ANOVA Age-sex class*	*ANOVA Interactions*
**GLUCOSE (g/L)**	*Pan troglodytes*	0.02 [0–1.00]	F_1,272_ = 1.45 P = 0.230	0.05 [0.00–1.00]	0.00 [0.00]	F_1,272_ = 0.00 P = 0.944	0.00 [0.00]	0.38 [0.00–1.00]	0.06 [0.00–1.00]	0.09 [0.00–1.00]	0.00 [0.00]	0.00 [0.00]	F_5,272_ = 1.12 P = 0.348	species*season: F_1,272_ = 0.00 P = 0.974; species*age/sex class: F_5,272_ = 0.82 P = 0.538; season*age/sex class: F_5,272_ = 0.62 P = 0.680
	*Gorilla gorilla*	0.15 [0.00–20.00]		0.18 [0.00–20.00]	0.07 [0.00–2.50]		0.00 [0.00]	0.38 [0.00–20.00]	0.00 [0.00]	0.19 [0.00–2.50]	0.06 [0.00–2.50]	0.05 [0.00–1.00]		
**PROTEIN (g/L)**	*Pan troglodytes*	0.14 [0.00–2.00]	**F_1,268_ = 6.65 P = 0.010**	0.16 [0.00–2.00]	0.12 [0.00–1.00]	F_1,268_ = 0.32 P = 0.572	0.15 [0.00–2.00]	0.13 [0.00–1.00]	0.07 [0.00–0.30]	0.20 [0.00–0.30]	0.19 [0.00–1.00]	0.00 [0.00]	F_5,268_ = 0.98 P = 0.430	species*season: F_1,268_ = 0.63 P = 0.426; species*age/sex class: F_5,268_ = 1.18 P = 0.316; season*age/sex class: F_5,268_ = 0.76 P = 0.581
	*Gorilla gorilla*	0.05 [0.00–0.30]		0.05 [0.00–0.30]	0.04 [0.00–0.20]		0.04 [0.00–0.30]	0.05 [0.00–0.30]	0.07 [0.00–0.30]	0.03 [0.00–0.20]	0.05 [0.00–0.30]	0.03 [0.00–0.20]		
**BLOOD (Ery/microL)**	*Pan troglodytes*	1.69 [0.00–80.00]	**F_1,270_ = 0.51 P = 0.474**	2.18 [0.00–80.00]	1.22 [0.00–80.00]	F_1,270_ = 0.00 P = 0.957	0.49 [0.00–10.00]	1.13 [0.00–10.00]	0.56 [0.00–10.00]	8.18 [0.00–80.00]	0.00 [0.00]	40.00 [0.00–80.00]	**F_5,270_ = 8.31, P<<0.001**	species*season: F_1,270_ = 0.18 P = 0.669 species*age/sex class **: F_5,270_ = 5.34 P<<0.001 ** season*age/sex class: ** F_5,270_ = 3.65 P = 0.003**
	*Gorilla gorilla*	3.39 [0.00–80.00]		2.64 [0.00–80.00]	5.31 [0.00–80.00]		0.00 [0.00]	2.31 [0.00–80.00]	4.09 [0–80]	12.31 [0–80]	2.93 [0.00–80.00]	5.56 [0.00–80.00]		
**LEUKOCYTES (Luc/microL)**	*Pan troglodytes*	15.51 [0.00–500.00]	F_1,362_ = 25.16 P<<0.001	24.04 [0.00–500.00]	7.09 [0.00–70.00]	F_1,256_ = 0.75 P = 0.386	17.03 [0.00–500.00]	21.23 [0.00–500.00]	6.39 [0.00–70.00]	6.00 [0.00–15.00]	8.67 [0.00–70.00]	0.00 [0.00]	**F_5,256_ = 2.22 P = 0.053**	species*season: F_1,256_ = 0.00 P = 0.949; species*age/sex class: F_5,256_ = 1.93 P = 0.090; season*age/sex class: F_5,256_ = 0.72 P = 0.61
	*Gorilla gorilla*	33.53 [0.00–125.00]		33.84 [0.00–125.00]	32.77 [0.00–125.00]		15.36 [0.00–70.00]	42.44 [0.00–125.00]	21.19 [0.00–125.00]	36.92 [0.00–125.00]	36.92 [0.00–125.00]	44.71 [0.00–125.00]		

In parenthesis the range of maximum and minimum values recorded. Columns named “Anova” show ANOVA's results of the three different factors tested: species, season and age/sex class. See text for details on statistical method.

## Discussion

This study is the first comparative study investigating temporal variation of the same clinical signs, urinary function variables and intestinal parasites, in chimpanzees and western gorillas. We found that both great ape populations experience comparable challenges with regard to general health even though chimpanzees revealed a higher occurrence of diarrhoea and wound records while western gorillas had marginally greater parasite diversity and higher prevalence and intensity of parasite and urinary infections.

### Ape population and time period differences

Among the clinical signs, the most common symptoms recorded in the two apes were respiratory symptoms (coughs and sneezes), corroborating evidence for respiratory infections being the major cause of disease in several chimpanzee communities and at least in mountain gorillas (reviewed in [85]). Ape population differences in the occurrence of diarrhoea and injuries supports our hypothesis that the greater level of social interactions within chimpanzee communities may increase the frequency of at least these two health parameters. However, the impact of human contacts on the frequency of diarrhoea cannot be ruled out in this study and further investigations are needed.

Some species of parasites and ciliates were found in both host populations (*Oesophagostomum sp.*, *Probstmayria gombensis*, *Bertiella studeri*, and *Troglodytella abrassarti*), while others were specific to one ape population (chimpanzee: small ciliates and *Strongyloides fulleborni*; western gorillas: *Mammomonogamus sp.*, *Protospirura muricola* and strongyles with small eggs different from *Strongyloides fullebornii*). As previously found in sympatric populations of these apes, western gorillas had an overall higher parasite prevalence, dominated by infections by *Oesophagostomum sp.* and other strongyle species, the most abundant parasites for both host species [6], [9], [86]–[89]. Prevalence and intensity of all strongyle infections (*Oesophagostomum sp.*, small egg strongyles for gorillas, and *Strongyloides fulleborni* for chimpanzees) were highly affected by the different study time periods. These patterns were dominated by higher western gorilla parasite infections during the dry period with respect to the wet period contrary to our predictions based on previous study investigating effects of seasonality on chimpanzee parasites ([6], [16], [71] but see [72]). In general, seasonality seemed less marked in the Ugandan forest ([78], Table 1), in chimpanzee annual diet [64], [66] and consequently in the prevalence and intensity of parasite infections. The more herbivorous diet and greater changes in the nutritional status of western gorillas during periods of fruit scarcity (dry season) may induce nutritional stress or simply impose greater physiological requirements for gorillas [69] which may in turn influence their susceptibility to parasites (e.g. strongyles species). This hypothesis is also supported by higher urinary tract infections in western gorillas, even though, as previously found in chimpanzees, among all the urinary parameters leukocyte presence (urinary infections) was the most common positive parameter for both ape populations (Table S1) [6], [57]. However, the absence of ketones in gorilla urine confirms that during periods of fruit scarcity energy and macronutrient intake did not fall below their nutritional needs, urinary ketones being indicators for increased fat metabolism (as shown in orang-utans during large seasonal changes in food availability [22], [67]–[69], [90]). Nevertheless, during the dry season the western gorilla study group consumed food that was lower in metabolizable energy, and alongside the lower frugivory and dietary diversity, likely experienced lower intake of certain vitamins [68]–[69], [91]. Future studies may investigate the role played by pre-ingestion selection of specific plants/compounds by chimpanzees (e.g. rough leaves swallowing never observed in the studied gorillas) that may efficiently maintain lower level of intestinal parasite infections in comparison to gorillas, whose higher fermentation ability may neutralize both harmful and beneficial compounds [32], [37], [38], [61], [63], [65], [92], [93], [94].

Our results on cestode presence in western gorillas show for the first time that lowland habitats may harbor adequate intermediate hosts (presumably oribatid mites) for the cestode life cycle which have only previously been found at high prevalence in mountain gorillas and thought to be absent or relatively scarce even in the studied population of western gorillas [70], [86], [87], [95], [96]. Our longer study period (including different time periods with meteorological variations, Table 1) may account for differences with previous studies particularly for western gorillas, for example the detection for the first time of an adult of *Protospirura muricola* in gorillas (but presumptive eggs in [95]), a nonpathogenic rodent parasite, causing severe disease in primates [97], [98].

Alternative hypotheses are also plausible for the observed differences in intestinal parasites between wet and dry periods of our study period. Our results may also reflect higher dry season transmission of nematodes (particularly strongyles) and possibly cestodes for both apes due to the life cycles of the parasites themselves and their availability in the habitat – even though, previous studies on chimpanzees have shown the opposite pattern (higher prevalence in the wet season: [6], [71], [16]). Furthermore, there may be greater ape-parasite contact during drier periods when apes rely more on herbivorous/terrestrial diet. For example, the gorilla study group visit clearings more frequently during the dry season (Masi, unpublished data, Bai Hokou, long term data), increasing contact with rivers/swamps which is favourable environment for strongyles [99]. This behaviour, together with the regular close proximity to all individuals within a cohesive gorilla group in contrast to the occasional proximity, albeit closer interactions, among individuals within a chimpanzee community [58]–[61] may also contribute to higher parasite prevalence in gorillas. In comparison to chimpanzees western gorillas are also into greater contact with the soil where strongyles eggs are abundant [99] since they frequently sleep on the ground in nests devoid of vegetation [100] and feed on fallen fruit or terrestrial feeding remains of other gorillas ([60], [101], Masi, unpublished data).

Among all the specific parasites found, the lungworm *Mammomonogamus sp.* was the only one occurring at higher prevalence during the wet period and, as in previous studies, it was absent in chimpanzees (e.g. [6], [16], [40], [86], [87]). The absence of this nematode in the neighboring gorilla populations suggest differences in parasite distribution rather than in host species susceptibility [86], [87], [95].

Entodiniomorph ciliates, found only in chimpanzee, were more abundant (prevalence and load) during the dry period, likely reflecting the need for higher fibre fermentation when fruit is scarce. The absence of entodiniomorph ciliates and the lower presence of *Troglodytella sp.* in gorillas contrast with previous findings from sympatric gorilla and chimpanzee populations that suggested ciliates are responsible for greater cellulose digestion in gorillas [86]. However, hydrolytic activities of intestinal bacteria were recently shown to play a more important role than ciliates for the digestion processes of great apes, thus explaining our results [102].

Compared to gorillas, chimpanzees had higher protein urine levels. In humans high levels of proteins in urine are associated with physical activity, fever, or urinary tract infections [103]. Even though gorillas had higher urinary infections, this result can be linked to the more frequent diarrhoea, wounds, and thirst symptoms (likely associated with fever episodes; Figure 1) recorded in chimpanzees.

### Age-sex inter-species comparison

As for other primates, young western gorillas appeared to be more susceptible to disease as infants and juveniles had higher intestinal parasite loads (all strongyles) with respect to the other age/sex classes and the chimpanzee population (e.g. [46]–[47], [87]). The high susceptibility of young individuals can have a major impact on the growth rate of the population. Moreover, during the dry season of the study period gorilla infants had a higher urinary erythrocyte concentration than all other age/sex classes (but subadult females). While reasons for the presence of urinary erythrocytes remains unknown in this study, this result strengthens our hypothesis of the higher impact of temporal climatic and dietary changes (i.e. during dry season) on western gorilla health since its influence appears particularly severe for the most vulnerable age class. In contrast to chimpanzees, alongside their habit of feeding on adult leftovers, infection transmission in western gorilla infants may also be increased by the consumption of food items that are rarely or never eaten by adults, including rotten fruit [61]. Higher urine blood content in subadult females may be explained by minor trauma to their vaginas caused by sexual swelling [57] and/or by the first rough copulations observed in chimpanzees and inexperienced subadult dyads for gorillas (Krief and Masi, unpublished data).

### Conclusions

Given that we present data only on one group of each ape species and one dry/wet season, this study could only ever provide preliminary results on temporal differences in infection rates between two ape populations. Further studies combining data on multiple seasons and sympatric chimpanzee and gorilla populations are needed to confirm these hypothesis and the observed seasonal patterns. In our study we cannot exclude geographical dissimilarity in parasite density and ecology may have played a role. However, given that other studies have shown that sympatric ape species and populations may also not share the same parasites [86], [87], [95], our findings of higher parasite and urine infections in western gorillas with respect to chimpanzees suggest a possible higher seasonal impact (e.g. dietary “stress”) on western gorilla health. Moreover, our alternative prediction concerning the higher complexity of social interactions (including social stress) in chimpanzees may be more important for other clinical signs such as the transmission of infections leading to diarrhoea and the infliction of wounds.

Western gorillas are critically endangered [54], [104] and recently have shown seriously declines showing low resilience in the face of high susceptibility to diseases such as Ebola hemorrhagic fever, even more so than sympatric chimpanzees [51], [55], [105]. Chimpanzees are endangered [104] and almost all monitored communities have been severely impacted by disease [85]. Health monitoring of non-human primates is of vital importance both for human health and species conservation enabling health assessments of endangered populations before and during outbreaks [1], [3]. Our results raise potential concerns for the future of western gorillas and chimpanzees despite their great dietary flexibility. Nutritional status and vulnerability to infectious disease of both species may be exacerbated by human encroachment on ape habitat and the potential loss of plant biodiversity: gorillas being more affected by seasonal nutrient stress and chimpanzees being dependant on a wide repertoire of plants to control infections [35–71–94]. Therefore, global changes (i.e. climate change, forest fragmentation and destruction) may intensify this impact on African great ape health, particularly for those populations who face larger seasonal fluctuations of the environment. However, results comparing different geographical areas need to be treated with caution and further investigations are imperative to assist prioritizing conservation efforts on more sensitive ape populations (i.e. living in habitat with larger seasonal fluctuations) in order to maintain population structure and demography and reduce habitat pressure.

## Supporting Information

Figure S1 **Monthly number of sampled identified individuals and, monthly average number of samples per individual.** See the text for number of individuals per age/sex classes per study group/community.
(TIF)

Table S1 Comparative summary of percentage of samples for each urine parameter for chimpanzees and western gorillas. Darker grey lines underline normal human range as provided by the Trade Mark (Bayer Multistix 10 SG), lighters grey indicates possible minor inconsistent clinical worries.
(DOC)
