# Supplementary figures and images for: Seasonal Effects on Great Ape Health: A Case Study of Wild Chimpanzees and Western Gorillas
Source: PLoS One. 2012 Dec 5;7(12):e49805. doi: 10.1371/journal.pone.0049805 (PMC3515584; doi:10.1371/journal.pone.0049805)

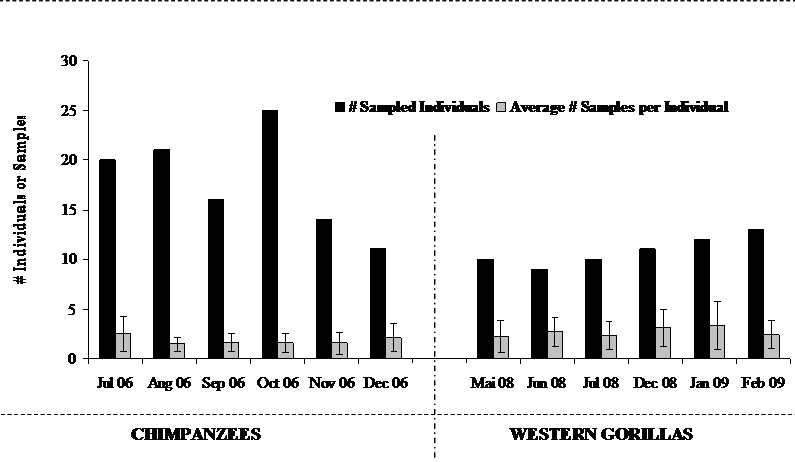

Supplement: Figure S1 — Monthly number of sampled identified individuals and, monthly average number of samples per individual. See the text for number of individuals per age/sex classes per study group/community. (TIF) [file pone.0049805.s001.tif]
