# Supplementary material for: Seasonal Effects on Great Ape Health: A Case Study of Wild Chimpanzees and Western Gorillas
Source: PLoS One. 2012 Dec 5;7(12):e49805. doi: 10.1371/journal.pone.0049805 (PMC3515584; doi:10.1371/journal.pone.0049805)
Supplement: Table S1 — Comparative summary of percentage of samples for each urine parameter for chimpanzees and western gorillas. Darker grey lines underline normal human range as provided by the Trade Mark (Bayer Multistix 10 SG), lighters grey indicates possible minor inconsistent clinical worries. (DOC) [file pone.0049805.s002.doc]

**Table S1. Comparative summary of percentage of samples for each urine parameter for chimpanzees and western gorillas.** Darker grey lines underline normal human range as provided by the Trade Mark (Bayer Multistix 10 SG), lighters grey indicates possible minor inconsistent clinical worries.

| **% URINE SAMPLES** | ***Pan troglodytes*** | ***Gorilla gorilla*** |  | **% URINE SAMPLES** | ***Pan troglodytes*** | ***Gorilla gorilla*** |  | **% URINE SAMPLES** | ***Pan troglodytes*** | ***Gorilla gorilla*** |
| --- | --- | --- | --- | --- | --- | --- | --- | --- | --- | --- |
| **Glucose (g/L) N** | 160 | 176 |  | **Proteins (g/L) N** | 157 | 176 |  | **Specific Gravity N** | 160 | 176 |
| Negative (<1) | 97 | 97 |  | Negative (<0.15) | 60 | 78 |  | Low (1.000-1.014) | 62 | 76 |
| Trace (1-2.4) | 3 | 1 |  | Trace (0.15-0.29) | 8 | 18 |  | Moderate (1.015-1.024) | 13 | 17 |
| Moderate (2.5-9) | 0 | 1 |  | Moderate (0.30-2.9) | 29 | 3 |  | High (≥1.025) | 25 | 7 |
| Severe (≥10) | 0 | 1 |  | Severe (≥3) | 33 | 0 |  | Unclear Result | 0 | 0 |
| Unclear Result | 0 | 0 |  | Unclear Result | 0 | 1 |  |  |  |  |
|  |  |  |  |  |  |  |  | **Nitrites N** | 157 | 176 |
| **Ketones (g/L) N** | - | 175 |  | **Bilirubin N** | 160 | 176 |  | Negative | 96 | 30 |
| Negative (<0.05) | - | 66 |  | Negative | 94 | 25 |  | Moderate | 4 | 43 |
| Trace (0.05-0.14) | - | 4 |  | Moderate (+) | 1 | 14 |  | Severe | 0 | 26 |
| Moderate (0.15-0.79) | - | 1 |  | Severe (++ and +++) | 3 | 5 |  | Unclear Result | 0 | 1 |
| Severe (≥0.8) | - | 0 |  | Unclear Result | 2 | 56 |  |  |  |  |
| Unclear Result | - | 29 |  |  |  |  |  | **Ph N** | 157 | 176 |
|  |  |  |  | **Blood (Ery/microL) N** | 160 | 175 |  | 5.0-6.9 | 21 | 36 |
| **Leukocytes (Leu/microL) N** | 157 | 177 |  | Negative (<10) | 92 | 88 |  | 7-7.9 | 8 | 5 |
| Negative (<15) | 68 | 20 |  | Non-Haemolyzed Trace (10-79) | 7 | 4 |  | ≥8 | 71 | 59 |
| Trace (≥15-69) | 22 | 35 |  | Non-Haemolyzed Moderate (≥80) | 1 | 2 |  | Unclear Result | 0 | 0 |
| Moderate (70-124) | 9 | 15 |  | Haemolyzed Trace (10-24) | 0 | 1 |  |  |  |  |
| Severe (≥125) | 1 | 2 |  | Haemolyzed Moderate (25-79) | 0 | 0 |  | **Urobilinogen (mg/dL) N** | 157 | 175 |
| Unclear Result | 0 | 28 |  | Haemolyzed Severe (≥80) | 0 | 1 |  | Normal * (0.2-1.9) | 97 | 99 |
|  |  |  |  | Unclear Result | 0 | 4 |  | Moderate (2-3.9) | 0 | 0 |
|  |  |  |  |  |  |  |  | Severe (≥4) | 1 | 0 |
|  |  |  |  |  |  |  |  | Unclear Result | 2 | 1 |
